# Directional coupling of slow and fast hippocampal gamma with neocortical alpha/beta oscillations in human episodic memory

**DOI:** 10.1073/pnas.1914180116

**Published:** 2019-10-09

**Authors:** Benjamin J. Griffiths, George Parish, Frederic Roux, Sebastian Michelmann, Mircea van der Plas, Luca D. Kolibius, Ramesh Chelvarajah, David T. Rollings, Vijay Sawlani, Hajo Hamer, Stephanie Gollwitzer, Gernot Kreiselmeyer, Bernhard Staresina, Maria Wimber, Simon Hanslmayr

**Affiliations:** ^a^School of Psychology, University of Birmingham, Birmingham B15 2TT, United Kingdom;; ^b^Centre for Human Brain Health, University of Birmingham, Birmingham B15 2TT, United Kingdom;; ^c^Complex Epilepsy and Surgery Service, Neuroscience Department, Queen Elizabeth Hospital, Birmingham B15 2TH, United Kingdom;; ^d^Epilepsy Center, Department of Neurology, University Hospital Erlangen, 91054 Erlangen, Germany

**Keywords:** episodic memory, neural oscillations, intracranial EEG, hippocampus, human

## Abstract

Episodic memories hinge upon our ability to process a wide range of multisensory information and bind this information into a coherent, memorable representation. On a neural level, these 2 processes are thought to be supported by neocortical alpha/beta desynchronization and hippocampal theta/gamma synchronization, respectively. Intuitively, these 2 processes should couple to successfully create and retrieve episodic memories, yet this hypothesis has not been tested empirically. We address this by analyzing human intracranial electroencephalogram data recorded during 2 associative memory tasks. We find that neocortical alpha/beta (8 to 20 Hz) power decreases reliably precede and predict hippocampal “fast” gamma (60 to 80 Hz) power increases during episodic memory formation; during episodic memory retrieval, however, hippocampal “slow” gamma (40 to 50 Hz) power increases reliably precede and predict later neocortical alpha/beta power decreases. We speculate that this coupling reflects the flow of information from the neocortex to the hippocampus during memory formation, and hippocampal pattern completion inducing information reinstatement in the neocortex during memory retrieval.

## Significance

Episodic memories detail our personally experienced past. The formation and retrieval of these memories have long been thought to be supported by a division of labor between the neocortex and the hippocampus, where the former processes event-related information and the latter binds this information together. However, it remains unclear how the 2 regions interact. We uncover directional coupling between these regions, with power decreases in the neocortex that precede and predict power increases in the hippocampus during memory formation. Fascinatingly, this process reverses during memory retrieval, with hippocampal power increases preceding and predicting neocortical power decreases. These results suggest a bidirectional flow of information between the neocortex and hippocampus is fundamental to the formation and retrieval of episodic memories.

**Fig. 1.**
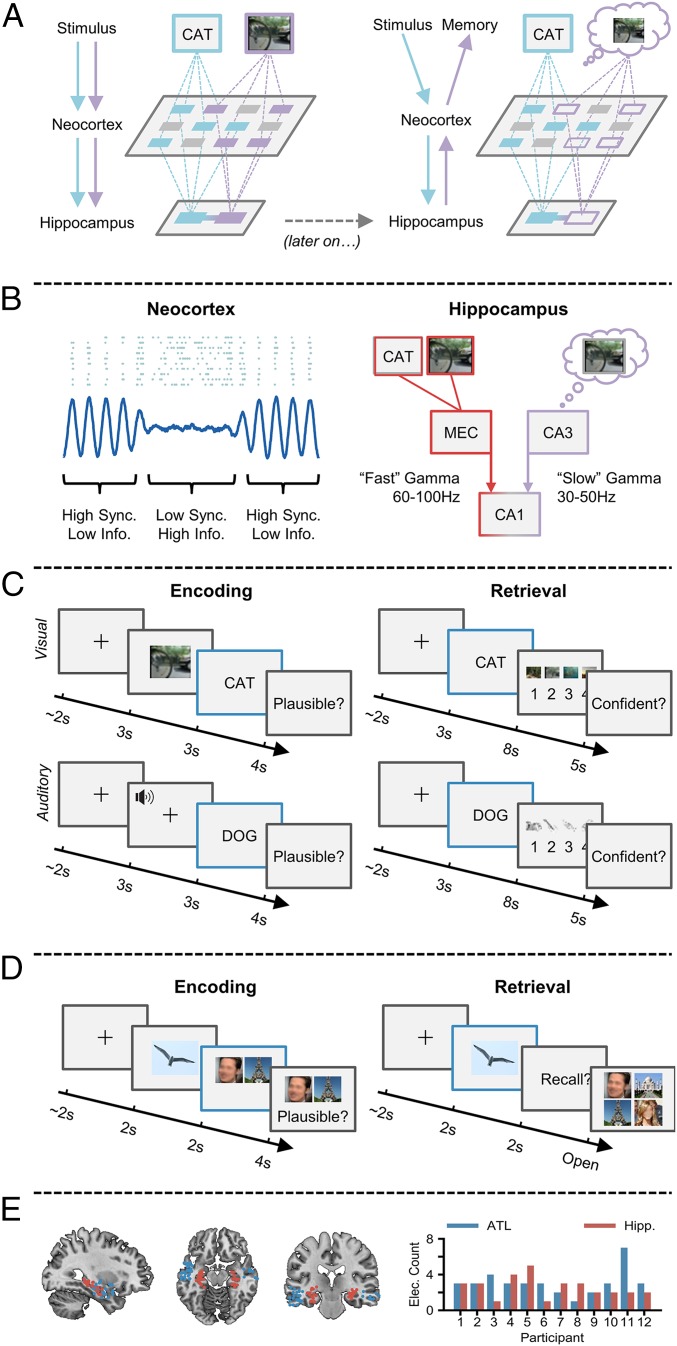
Synchronization–desynchronization framework. (*A*) Incoming stimuli are independently processed by relevant sensory regions of the neocortex (*Left*), and then passed on to the hippocampus where they are bound together. At a later stage (*Right*), a partial cue reactivates the hippocampal associative link, which in turn reactivates neocortical patterns coding for the memory representation, giving rise to conscious recollection. (*B*) Reduced oscillatory synchronization (blue line) within the neocortex allows individual neurons (blue dots) to fire more freely and create a more flexible neural code. Fast gamma activity allows the transfer of neocortical information to the hippocampus by boosting connectivity between the entorhinal cortex (MEC) and CA1. Slow gamma enhances retrieval by boosting connectivity between CA3 and CA1, allowing reinstated memories to be passed to the neocortex. (*C*) During encoding, participants are tasked with forming an associative link between a life-like dynamic stimulus (either a video or sound) and a subsequent verbal stimulus. During retrieval, participants are presented with verbal stimuli from the previous encoding block and asked to retrieve the associated dynamic stimulus. Electrophysiological analysis was conducted during the presentation of the verbal stimulus at encoding and retrieval (blue outline). (*D*) During encoding, participants are tasked with forming an associative link between an object, a face, and a scene. During retrieval, participants are presented with the object and asked to retrieve the associated face and scene. Electrophysiological analysis was conducted during the presentation of the verbal stimulus at encoding and retrieval (blue outline). (*E*) Plot of each electrode location (*Left*; red represents hippocampal electrode; blue represents the ATL). Bar plot (*Right*) depicts the number of electrodes for each participant.

An episodic memory is a highly detailed memory of a personally experienced event (1, 2). The formation and retrieval of such memories hinge upon 1) the processing of information relevant to the event, and 2) the binding of this information into a coherent episode. A recent framework (3) and computational model (4) suggest that the former of these processes is facilitated by the desynchronization of neocortical alpha/beta oscillatory networks (8 to 20 Hz; reflected in decreases in oscillatory power) (5), while the latter is facilitated by the synchronization of hippocampal theta and gamma oscillations (3 to 7 Hz; 40 to 100 Hz; reflected in increases in oscillatory power) (6, 7) (Fig. 1*A*). Critically, the framework posits that these 2 mechanisms need to cooperate, as an isolated failure of either of these mechanisms would produce the same undesirable outcome: an incomplete memory trace. Here, we test this framework and uncover evidence of an interaction between neocortical desynchronization and hippocampal synchronization during the formation and retrieval of human episodic memories. In addition, we demonstrate that distinct hippocampal gamma frequencies contribute to memory formation and retrieval, with “fast” gamma facilitating encoding and “slow” gamma facilitating retrieval.

Within the neocortex, desynchronized alpha/beta activity is thought to facilitate information processing (5). This hypothesis is based on the principles of information theory (8), which proposes that a system of unpredictable states (e.g., desynchronized neural activity, where the firing of one neuron is not predictive of the firing of another; see ref. 5 for details) is optimal for information coding (Fig. 1*B*). Neural desynchronization in humans is most often measured by a decrease in oscillatory power, as a strong correlation exists between neural synchronization and power (9) (though this link is strictly correlative). In support of the information-via-desynchronization hypothesis, many studies have observed neocortical alpha/beta power decreases during successful episodic memory formation (10–18) and retrieval (19–24). For example, neocortical alpha/beta power decreases scale with the depth of semantic processing during episodic memory formation (18). Critically, synchronizing alpha/beta rhythms via repetitive transcranial magnetic stimulation impairs both episodic memory formation and retrieval, suggesting that alpha/beta desynchronization plays a causal role in these processes (20, 25). In conjunction, these studies suggest that neocortical alpha/beta desynchronization underpins the processing of event-related information, allowing for the formation and later recollection of highly detailed episodic memories.

Within the hippocampus, synchronized gamma activity (30 to 100 Hz) is thought to be critical in the binding of event-related information, and the later retrieval of this information when prompted by a cue (6, 7, 26, 27). Entraining neurons to rhythms of ∼60 Hz (i.e., a fast gamma oscillation) allows for spike timing-dependent plasticity (STDP; a form of long-term potentiation) to occur (28), which strengthens synaptic connections between hippocampal neurons. As such, an increase in hippocampal fast gamma activity (60 to 100 Hz) may be a proxy for STDP (29, 30), and therefore representational binding. In contrast, a slower hippocampal gamma rhythm (30 to 50 Hz) has been proposed to facilitate memory retrieval (7, 31, 32). Slow gamma activity originates from the CA3 subfield of the hippocampus and may play a pivotal role in pattern completion (33, 34). The tradeoff in amplitude between these 2 gamma oscillations is thought to dictate whether encoding or retrieval takes place (35). Evidence suggests that periods of increased fast gamma activity enhance connectivity between CA1 and the entorhinal cortex (31, 36) (allowing information to flow into the hippocampus; Fig. 1*B*) and aid representational binding through STDP (28, 30). Meanwhile, periods of enhanced slow gamma activity see an increase in connectivity between CA1 and CA3 (allowing for the transfer of completed memory pattern into the neocortex; Fig. 1*B*) (31, 36). In conjunction, these findings and theories would suggest that fast and slow gamma rhythms differentially support the hippocampal ability to associate and reactivate discrete elements of an episodic memory.

Here, we investigated the coordination between alpha/beta power decreases in the anterior temporal lobe and gamma power increases in the hippocampus during episodic memory formation and retrieval. Specifically, we tested 4 central hypotheses derived from a series of conceptual frameworks, computational models, and rodent studies: 1) fast gamma oscillations (60 to 100 Hz) will support encoding while slow gamma oscillations (30 to 45 Hz) will support memory retrieval (7, 31); 2) neocortical power decreases [reflecting information processing (5)] and hippocampal power increases [reflecting representational binding (6, 7, 26, 27)] will accompany episodic memory formation and retrieval when contrasted against memories that were not successfully encoded/retrieved; 3) neocortical power decreases will precede hippocampal power increases during memory formation (reflecting information processing preceding representational binding); and 4) hippocampal power increases will precede neocortical power decreases during retrieval (reflecting pattern completion preceding information reinstatement) (3, 4).

Twelve patients implanted with stereotactic electroencephalogram (EEG) electrodes for the treatment of medication-resistant epilepsy completed 1 of 2 associative memory tasks (Fig. 1 *C* and *D*; *n* = 7 in task 1; *n* = 5 in task 2). In task 1, they related life-like videos or sounds to words that followed. Following a short distractor task, participants attempted to recall the previously presented videos/sounds using the words as cues. In task 2, they related an object to pairs of visual stimuli that followed (face–place, face–face, or place–place). Following a short distractor task, participants attempted to recall both stimuli, using the object as a cue. While external stimulation is different between the 2 tasks, the underlying cognitive and neural processes relating to our hypotheses are consistent: Both tasks require sensory processing followed by representational binding during memory formation, and hippocampal pattern competition prior to neocortical reinstatement during memory retrieval. As such, the data from the 2 tasks were pooled together for analysis. We conducted these analyses in 2 regions of interest (ROIs) (Fig. 1*E*): the hippocampus (a hub for representational binding) and the anterior temporal lobe [ATL; a hub for semantic-based information processing (37)]. Foreshadowing the results below, we show that ATL alpha/beta power decreases precede hippocampal fast gamma power increases during successful memory formation, and that hippocampal slow gamma power increases precede ATL alpha/beta power decreases during successful memory retrieval.

## Results

### Behavioral Results.

Participants, on average, recalled 47.9% of all pairs in the first task, a percentage much greater than what would be expected by chance (25%). When breaking trials down by modality, participants recalled 52.7% of video–word pairs and 45.9% of sound–word pairs. An independent-samples *t* test (only a subset of participants completed both variants of the task) revealed no significant difference in memory performance for video–word and sound–word pairs (*P* > 0.5, *d* = 0.275). As there was no apparent difference in memory performance between the 2 trial types and electrode contacts were not located in anatomical regions that should respond uniquely to one of these sensory modalities, trials involving video–word and sound–word pairs were combined for all further analyses. In the second task, participants recalled both associated items on 66.2% of trials—a percentage much greater than what would be expected by chance (16.7%; where the probability of selecting the first item correctly is 50% and the probability of selecting the second item correctly is 33%, making the joint probability 50% × 33% ≈ 16.6%).

### Distinct Oscillatory Signatures Exist in the Neocortex and Hippocampus.

**Fig. 2.**
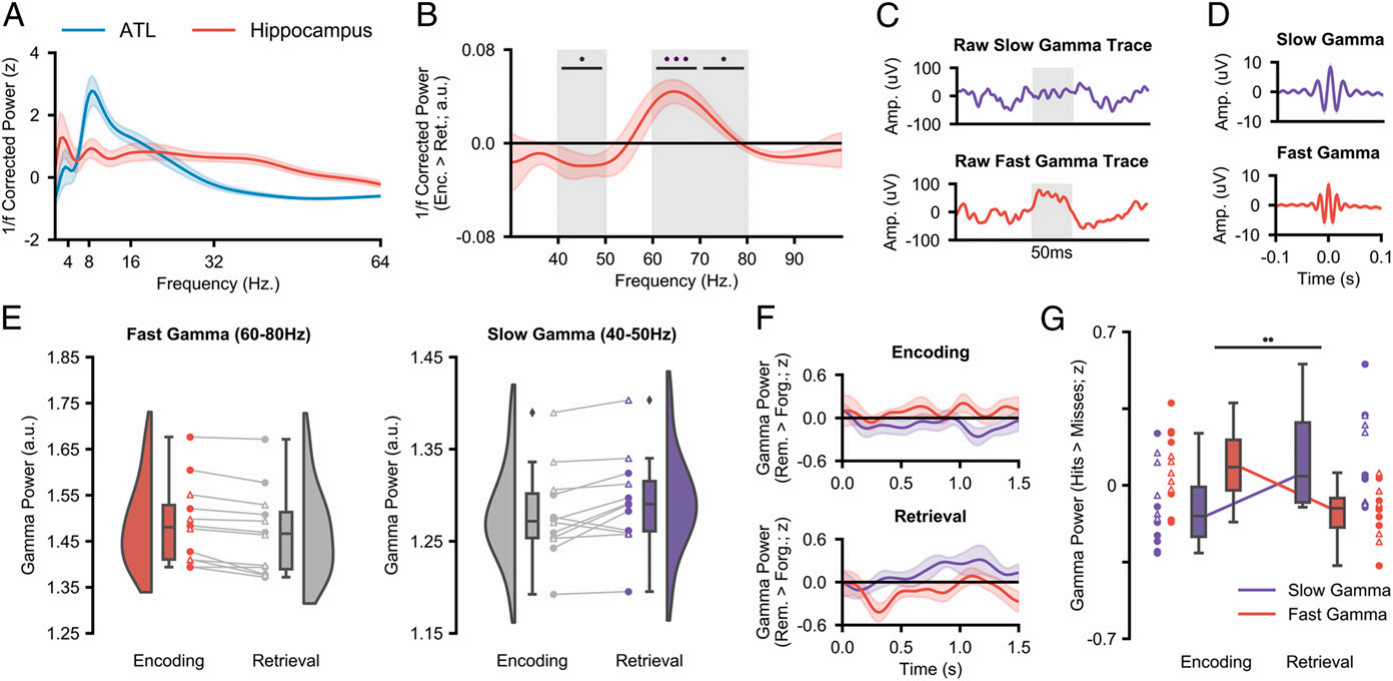
Hippocampal gamma activity during encoding and retrieval. (*A*) The mean 1/*f*–corrected power spectrum (with shaded SEM) across all encoding and retrieval trials reveals theta and gamma peaks in the hippocampus and an alpha/beta peak in the ATL. (*B*) The mean difference in gamma power (with shaded SEM) between encoding and retrieval reveals a peak in encoding-related, fast gamma at 60 to 80 Hz and a peak in retrieval-related, slow gamma at 40 to 50 Hz (*P_fdr_ < 0.05, ***P_fdr_ < 0.001). (*C*) Raw slow gamma signal during retrieval (*Top*) and fast gamma signal during encoding (*Bottom*) from a hippocampal contact of participant 1. The shaded gray regions indicate a period of 50 ms. (*D*) Mean peak-locked averaged signal across participants for slow (*Top*) and fast (*Bottom*) gamma (with shaded SEM). (*E*) Raincloud plots depicting the difference in fast (*Left*) and slow (*Right*) gamma power between encoding and retrieval. Colored circles represent participants who took part in experiment 1. Uncolored triangles represent participants who took part in experiment 2. (*F*) Time series of slow (in purple) and fast (in red) memory-related gamma power for encoding and retrieval. (*G*) Interaction between fast and slow gamma activity during encoding and retrieval. Encoding sees a relative increase of memory-related fast gamma power, while retrieval sees a relative increase of memory-related slow gamma power (**P_fdr_ < 0.01).

We first sought to empirically define the peak frequencies in our 3 ROIs. Broadband spectral power (1 to 100 Hz) was computed using 5-cycle wavelets across a 1,500-ms window starting at the onset of the verbal stimulus (at encoding and retrieval). The data were then *z*-transformed across trials to facilitate comparison across participants, and the 1/*f* component was subtracted from the data (38–40) to attenuate broadband noise (see *Methods* for details). Subsequently, the resulting power spectra were collapsed over time and trials, and split into hippocampal and neocortical ROIs. Across participants, a slow-theta peak could be observed in the hippocampus at ∼2.5 Hz and an alpha/beta peak could be observed in the 2 neocortical regions between 8 and 20 Hz (Fig. 2*A*). We defined the peak frequency of each ROI for each participant individually and conducted all subsequent analyses on these peak frequencies (see *SI Appendix*, Table S1 for individual peak frequencies).

### Distinct Hippocampal Gamma-Band Frequencies Underlie Encoding and Retrieval Processes.

We then investigated whether distinct gamma-frequency bands support encoding and retrieval processes (7, 31). To test this, the broadband hippocampal gamma power (30 to 100 Hz) for successfully remembered pairs at encoding and retrieval was calculated and contrasted in a group-level, nonparametric permutation test. Fast hippocampal gamma frequencies (60 to 80 Hz) exhibited significantly greater power during encoding, relative to retrieval, trials (60 to 70 Hz, *P*_fdr_ = 0.001, *d* = 1.308; 70 to 80 Hz, *P*_fdr_ = 0.020, *d* = 0.947; Fig. 2 *B–E*). In contrast, slow hippocampal gamma frequencies (40 to 50 Hz) exhibited greater power during retrieval, relative to encoding trials (*P*_fdr_ = 0.023, *d* = 0.754). No significant difference between encoding and retrieval could be observed during the epochs of forgotten stimuli (*SI Appendix*, Fig. S1). Peak fast and slow gamma frequencies for each participant were derived from the “encoding vs. retrieval” contrast and used in all subsequent analyses (see *Methods* for details; see *SI Appendix*, Table S1 for individual peak frequencies). These findings suggest that 2 functionally relevant gamma-band oscillations relate to episodic memory formation and retrieval in humans.

To rule out the possibility that the difference in fast/slow gamma was driven by the 1/*f* slope and/or its removal, the beta weights describing the 1/*f* slope at encoding and retrieval were extracted and averaged across time, electrodes, and trials. These weights were then contrasted between encoding and retrieval in a group-level, nonparametric permutation test. This test revealed no significant difference in the beta weights for remembered items (*P* = 0.198) or for forgotten items (*P* = 0.246), suggesting the distinction in gamma rhythms between encoding and retrieval was not driven by differences in the 1/*f* slope.

### Hippocampal Gamma Power Increases Track the Successful Formation and Retrieval of Episodic Memories.

To examine how memory-related fluctuations in fast and slow gamma power differentially contribute to episodic memory encoding and retrieval, we conducted a group-level, nonparametric, permutation-based, 2 × 2 repeated-measures ANOVA that investigated the influence of the factors “gamma frequency” (fast vs. slow) and “memory operation” (encoding vs. retrieval) on memory-related power (remembered > forgotten) collapsed across time. We anticipated an interaction whereby fast gamma selectively supports successful memory formation and slow gamma selectively supports successful memory retrieval. Group analysis revealed a significant interaction (*P* = 0.003, partial η^2^ = 0.294; Fig. 2*G*), indicating that fast and slow gamma exhibited dissimilar memory-related power fluctuations during encoding and retrieval. These results demonstrate that 2 functionally distinct gamma-band oscillations support the successful formation and retrieval of episodic memories in humans.

Analysis of the power time series showed that the opposing effects of fast and slow gamma were particularly prominent during retrieval. When successfully recalling a stimulus, a rapid decrease in fast gamma power was observed (200 to 400 ms, *P*_fdr_ = 0.025, *d* = 0.862; Fig. 2*F*), followed by an increase in slow gamma power (800 to 1,000 ms, *P*_fdr_ = 0.007, *d* = 1.177; Fig. 2*F*), relative to stimuli that were not recalled. Perplexingly, a similar effect was not observed during encoding, even though the time series of the 2 gamma bands trend in the correct directions (i.e., an increase in fast gamma and a decrease in slow gamma; Fig. 2*F*). As will be revealed later, this absence may be driven by the fact that gamma power changes are not time-locked to stimulus onset during encoding but rather the neocortical power decreases that precede hippocampal activity.

### Neocortical Alpha/Beta Power Decreases Track the Successful Formation and Retrieval of Episodic Memories.

**Fig. 3.**
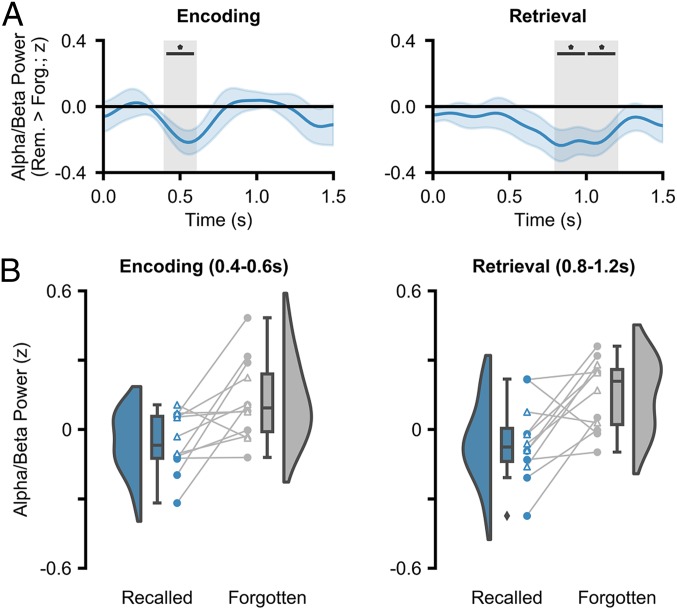
ATL alpha/beta activity during encoding and retrieval. (*A*) Time series of memory-related alpha/beta power for encoding and retrieval. In both cases, decreases in alpha/beta power relate to greater memory (*P_fdr_ < 0.05). (*B*) Raincloud plots depicting the difference in alpha/beta power between remembered and forgotten items. Colored circles represent participants who took part in experiment 1. Uncolored triangles represent participants who took part in experiment 2.

We then investigated whether neocortical alpha/beta power decreases accompany the successful encoding and retrieval of episodic memories. Peak alpha/beta power was computed across a 1,500-ms window commencing at stimulus onset. As above, the 1/*f* characteristic was subtracted, attenuating broadband noise (41, 42). The alpha/beta power was *z*-transformed across the entire session for each electrode–frequency pair separately, smoothed to attenuate trial-by-trial variability in temporal/spectral responses (*Methods*), and split into “hits” and “misses” for contrasting. A group-level, nonparametric permutation test revealed a significant decrease in ATL alpha/beta power during encoding (*P*_fdr_ = 0.035, *d* = 0.858; 400 to 600 ms after stimulus onset; Fig. 3) for remembered stimuli relative to forgotten stimuli. During retrieval, a group-level permutation test revealed a significant decrease in ATL alpha/beta power (800 to 1,000 ms, *P*_fdr_ = 0.042, *d* = 0.777; 1,000 to 1,200 ms, *P*_fdr_ = 0.039, *d* = 0.849; Fig. 3) for remembered stimuli relative to forgotten stimuli. These results reproduce earlier findings of neocortical alpha/beta power decreases during the encoding (10–18) and retrieval (19–24) of human episodic memories.

### Hippocampal Gamma Power Increases and Neocortical Alpha/Beta Power Decreases Cooperate during the Encoding and Retrieval of Human Episodic Memories.

So far, we have demonstrated that both neocortical alpha/beta power decreases and hippocampal fast and slow gamma power increases arise during episodic memory processes. Critically, however, the synchronization/desynchronization framework (3) would predict that these 2 markers correlate in such way that neocortical power decreases precede hippocampal power increases during encoding while hippocampal power increases precede neocortical power decreases during retrieval. Such a hypothesis can be tested through the use of cross-correlation, where the time series of neocortical alpha/beta power is offset relative to the time series of hippocampal gamma power in an attempt to identify at what time lag the 2 time series most strongly correlate. A negative lag indicates that early neocortical signals correlate with late hippocampal signals, while a positive lag indicates that early hippocampal signals correlate with late neocortical signals. Like traditional correlations, a negative correlation (from here on termed “anticorrelation”) indicates an increase in one metric is accompanied by a decrease in the other.

**Fig. 4.**
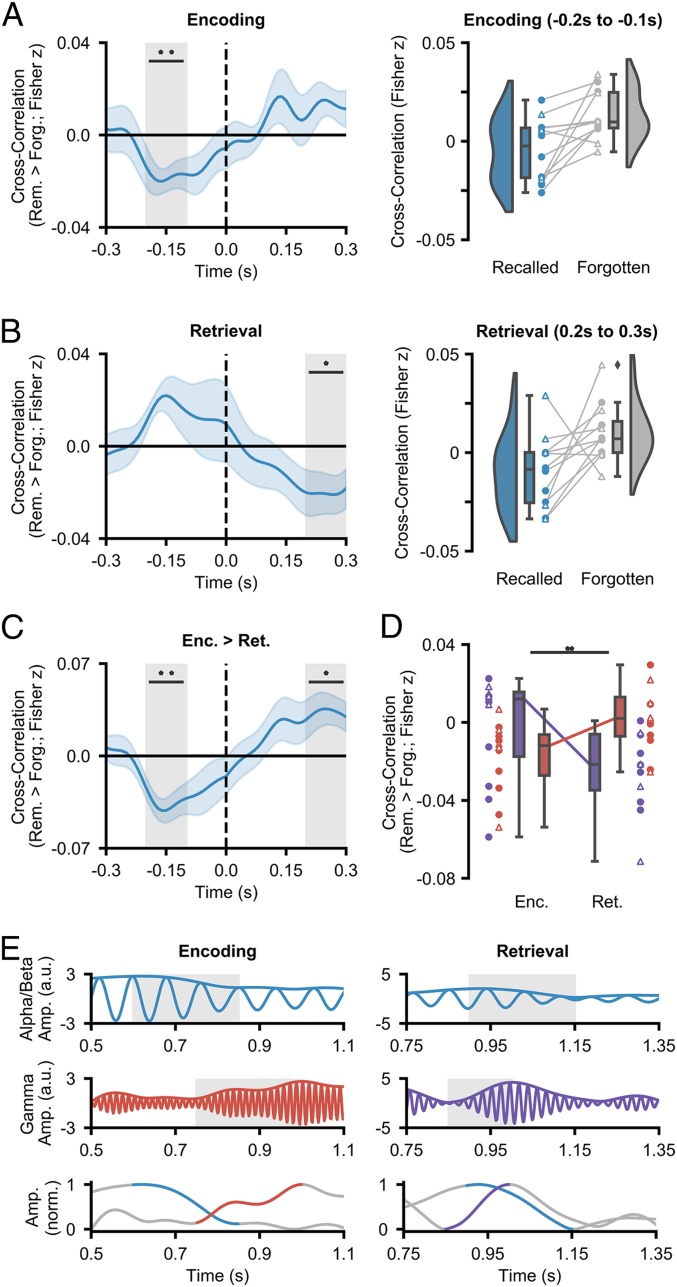
Hippocampal–neocortical time-series cross-correlations. (*A*) Mean cross-correlation (with shaded SEM; *Left*) between the hippocampal fast gamma power and ATL alpha/beta power during encoding (**P_fdr_ < 0.01). ATL power decreases precede hippocampal fast gamma power increases. Raincloud plot (*Right*) depicts the difference in cross-correlation between remembered and forgotten items. Colored circles represent participants who took part in experiment 1. Uncolored triangles represent participants who took part in experiment 2. (*B*) Mean cross-correlation (with shaded SEM; *Left*) between the hippocampal slow gamma power and ATL alpha/beta power during retrieval (*P_fdr_ < 0.05). Hippocampal slow gamma power increases precede ATL alpha/beta power decreases. Raincloud plot (*Right*) depicts the difference in cross-correlation between remembered and forgotten items. Colored circles represent participants who took part in experiment 1. Uncolored triangles represent participants who took part in experiment 2. (*C*) The contrast of cross-correlation activity between encoding and retrieval (*P_fdr_ < 0.05, **P_fdr_ < 0.01). (*D*) Mean cross-correlation between neocortical alpha/beta power and hippocampal gamma power (slow in purple; fast in red; with SEM) as a function of memory operation (*Top*, subject-level; *Bottom*, electrode pair-level). A repeated-measures ANOVA reveals an interaction between hippocampal gamma frequency and memory task when predicting memory-related hippocampal–neocortical cross-correlation (**P < 0.01). (*E*) Filtered single-trial traces at encoding (*Left*) and retrieval (*Right*) in the ATL (*Top*) and hippocampus (*Middle*). The envelopes of these traces are plotted (*Bottom*). During encoding, a reduction in ATL alpha/beta activity precedes an increase in hippocampal fast gamma power. During retrieval, an increase in hippocampal slow gamma power precedes a decrease in ATL alpha/beta activity.

At encoding, we hypothesized that the degree of neocortical power decreases can predict the degree of hippocampal gamma power increases (i.e., a negative lag anticorrelation). On a cognitive level, this would signify information processing within the neocortex preceding representational binding in the hippocampus. The cross-correlation was computed for every trial, and the memory-related difference was calculated by subtracting the mean cross-correlation across forgotten items from the mean cross-correlation across remembered trials. By calculating the memory-related difference, any correlation between the 2 time series that is driven by shared noise (originating from a shared reference) is removed, as this reference-related correlation is consistent across remembered and forgotten trials (additional analysis in *SI Appendix* confirms that shared reference activity does not account for the observed effects reported here). Furthermore, the memory-related difference highlights memory-specific dynamics in neocortical–hippocampal links, rather than general, memory-unspecific connectivity. In line with our hypothesis, later remembered items showed a significant anticorrelation at a negative lag between ATL alpha/beta power and hippocampal fast gamma power relative to later forgotten items (*P*_fdr_ = 0.006, *d* = 0.961; see Fig. 4*A* for a difference line plot). This cross-correlation suggests that alpha/beta power decreases precede fast gamma power increases by ∼100 to 200 ms. No correlation was observed between ATL alpha/beta power and hippocampal slow gamma power at any lag. These results indicate that a unique connection exists between the ATL and the hippocampus during episodic memory formation, where ATL power decreases precede hippocampal fast gamma power increases.

It is worth noting that while the absolute magnitude of the Fisher *z*-transformed correlation coefficients is small, one should exercise caution in interpreting such a value. As we focus on the difference in the ATL–hippocampal cross-correlation for remembered and forgotten items, we only probe the fraction of the total cross-correlation that can be explained by cognition and not that which can be accounted for by numerous undefinable variables (e.g., measurement noise, placing of electrodes, choice of reference, resting connectivity). As such, it is better to consider the variance in cross-correlation across participants. Here, the variance is minimal and hence returns a small *P* value (*P*_fdr_ = 0.006) and a large effect size (*d* = 0.961), indicating that ATL alpha/beta power decreases precede hippocampal fast gamma power increases reliably across participants.

We then investigated whether this relationship reverses during episodic memory retrieval (i.e., hippocampal power increases precede neocortical power decreases). On a cognitive level, this would represent pattern completion in the hippocampus preceding information reinstatement in the neocortex. To test this, we repeated the cross-correlation analysis in the same manner as above for epochs covering the presentation of the retrieval cue and then calculated the memory-related difference by subtracting the mean cross-correlation across forgotten items from the mean cross-correlation across remembered trials. Relative to forgotten items, remembered items showed a significant anticorrelation at a positive lag between ATL alpha/beta power and hippocampal slow gamma power (*P*_fdr_ = 0.037, *d* = 0.731; Fig. 4*B*), where an increase in hippocampal gamma power preceded a decrease in ATL alpha/beta power by 200 to 300 ms. No correlation was observed between ATL alpha/beta power and hippocampal fast gamma power at any lag. These results indicate that hippocampal slow gamma power increases precede ATL alpha/beta power decreases during the retrieval of episodic memories—a reversal of the dynamic observed during episodic memory formation.

We then examined how the neocortical–hippocampal dynamics differed between encoding and retrieval. To this end, the subsequent memory effect (SME; remembered minus forgotten cross-correlation at encoding) for ATL alpha/beta power and hippocampal fast gamma power was contrasted with the retrieval success effect (RSE; remembered minus forgotten cross-correlation at retrieval) for ATL alpha/beta power and hippocampal slow gamma power in a group-level, nonparametric, permutation test. This revealed an interaction whereby ATL power decreases preceded hippocampal power increases during encoding (*P*_fdr_ = 0.005, *d* = 1.181; 100 to 200 ms) but hippocampal power increases preceded ATL power decreases during retrieval (*P*_fdr_ = 0.025, *d* = 0.855; 200 to 300 ms) (Fig. 4*C*). These results support those reported in the previous 3 paragraphs: 1) ATL alpha/beta power decreases precede hippocampal fast gamma power increases during episodic memory formation, and 2) hippocampal slow gamma power increases precede ATL alpha/beta power decreases during episodic memory retrieval.

Lastly, we examined whether the fast gamma effect was specific to encoding and the slow gamma effect was specific to retrieval. To this end, we conducted a nonparametric, permutation-based, 2 × 2 repeated-measures ANOVA (memory operation × gamma frequency), taking encoding-related activity from the −200- to −100-ms time bin and retrieval-related activity from the 200- to 300-ms time bin. Analysis revealed a significant interaction between the 2 factors (*P* = 0.001; partial η^2^ = 0.172). The interaction (as pictured in Fig. 4*D*) suggests that the hippocampal fast gamma power negatively cross-correlated with ATL alpha/beta power to a greater degree than hippocampal slow gamma power during encoding, while the hippocampal slow gamma power negatively cross-correlated with ATL alpha/beta power to a greater degree than hippocampal fast gamma power during retrieval.

Notably, these effects cannot be explained by any epileptic activity such as IEDs (interepileptical discharges) traveling between the cortex and hippocampus. IEDs are broadband, so one may expect that IEDs that are temporally correlated across regions may give rise to spurious coupling between frequency bands. While certainly true, this cannot explain the effects observed here for 2 reasons. 1) Our findings are bidirectional—there would need to be pathological activity generated in both the ATL and the hippocampus to produce such bidirectional hippocampal–cortical interactions, where IEDs generated in the ATL travel to the hippocampus to produce the encoding effect, and IEDs generated in the hippocampus travel to the ATL to produce the retrieval effect. None of the patients who took part in the experiment had pathological tissue in both the ATL and the hippocampus, so the IED confound explanation cannot explain the directionality of our effect. 2) IEDs are broadband, yet our effects are narrowband. During encoding, we observe the cross-correlation between neocortical alpha/beta and hippocampal fast gamma, but importantly not neocortical alpha/beta and hippocampal slow gamma. Any IED-induced broadband artifact would inherently yield cross-correlations with alpha/beta power and both gamma bands, and not within 1 singular band. Complementary quantitative analysis to support this conclusion can be found in *SI Appendix*.

## Discussion

To successfully encode and recall episodic memories, we must be capable of 1) representing detailed multisensory information, and 2) binding this information into a coherent episode. Numerous studies have suggested that these 2 processes are accomplished by neocortical desynchronization (as measured by decreases in oscillatory power) and hippocampal synchronization (as measured by increases in fast and slow oscillatory gamma power), respectively (3, 5, 7, 26). Here, we show that these 2 processes coexist and interact. During successful episodic memory formation, alpha/beta power decreases in the anterior temporal lobe reliably precede fast hippocampal gamma power increases (60 to 80 Hz) by 100 to 200 ms. In contrast, slow hippocampal gamma power increases (40 to 50 Hz) precede alpha/beta power decreases by 200 to 300 ms during successful episodic memory retrieval. These findings demonstrate that the interaction between neocortical alpha/beta power decreases and hippocampal power increases in distinct, functionally relevant gamma rhythms underpins the formation and retrieval of episodic memories.

Our central finding demonstrates that ATL alpha/beta power decreases and hippocampal fast and slow gamma power increases interact during the formation and retrieval of episodic memories, respectively. This result draws together a multitude of conflicting studies, some of which indicate that synchronization benefits memory (e.g., refs. 43–45) and others which indicate that desynchronization benefits memory (e.g., refs. 13, 24, and 46), and provides a possible empirical resolution to the so-called synchronization–desynchronization conundrum (3). These findings are in line with previous observations demonstrating that hippocampal gamma power increases precede hippocampal alpha power decreases during associative memory retrieval (47). However, we also show that this sequence reverses during encoding, and that these 2 mechanisms interact across brain regions (via simultaneous hippocampal–neocortical recordings unavailable to ref. 47). We speculate that the delay in hippocampal response relative to ATL alpha/beta power decreases during encoding reflects the need for the ATL to process semantic details prior to the hippocampus binding this information into a coherent representation of the event (26, 27). In contrast, we posit that the ATL delay in response relative to hippocampal gamma power increases during retrieval reflects the need for the hippocampal representational code to be reactivated prior to reinstating highly detailed stimulus-specific information about the event (48). Anatomically speaking, this reciprocal communication may be facilitated by the “direct intrahippocampal pathway”—a route with reciprocal connections between the ATL and hippocampus via the entorhinal cortex (49, 50). These anatomical connections would allow the ATL and hippocampus to cooperate during episodic memory formation and retrieval, facilitating the flow of neocortical information into the hippocampus during encoding and the propagation of hippocampal retrieval signals into the neocortex during retrieval.

We also found distinct gamma rhythms supporting human episodic memory formation and retrieval (7, 35). Specifically, we found greater fast gamma oscillatory activity (60 to 80 Hz) during encoding and greater slow gamma oscillatory activity (40 to 50 Hz) during retrieval, generalizing earlier rodent findings (e.g., ref. 31) to humans. We uncovered similar distinctions in fast and slow gamma-band activity when investigating memory-related changes in power and neocortical–hippocampal cross-correlations, providing additional evidence for such a distinction. Earlier rodent studies have suggested that the distinction between the 2 gamma bands reflects a difference in CA1 coupling (31); fast gamma oscillations support CA1–entorhinal cortex coupling, facilitating the transfer of information into the hippocampus, while slow gamma oscillations support CA1–CA3 coupling, facilitating the reactivation of stored information. We speculate that these patterns of connectivity extrapolate to humans and explain the observed differences in gamma frequency relating to episodic memory formation and retrieval. In sum, our results suggest that fast and slow gamma activity relates to distinct processes in the successful formation and retrieval of episodic memory.

In combination, the cross-correlation and gamma-band analyses produce a detailed picture of information flow during episodic memory formation and retrieval. Based on earlier frameworks (3, 7) and models (4), we postulate that the link between neocortical alpha/beta power decreases and hippocampal fast gamma power increases during memory formation reflects the flow of semantic information (processed in the ATL) to the entorhinal cortex (27) via the direct intrahippocampal pathway (49, 50), where fast gamma synchronicity between the entorhinal cortex and CA1 passes this information on to the hippocampus (31, 51). In contrast, the link between hippocampal slow gamma power increases and neocortical alpha/beta power decreases during memory retrieval reflects the flow of reactivated representational codes from CA3 to CA1 [via slow gamma synchronicity (31, 51)], which propagates out into the neocortex (48) via reciprocal connections in the direct intrahippocampal pathway, reinstating semantic details in the desynchronized ATL. However, future research with direct recordings from these hippocampal subregions in humans is needed to empirically test this proposed flow of information during episodic memory formation and retrieval.

Two questions remain, however. First, do similar bidirectional streams of information flow exist between the hippocampus and other neocortical regions? As it was not medically necessary, electrode coverage did not expand to every neocortical region linked to episodic memory. Therefore, we could not test this theory. We speculate, however, that similar bidirectional links do exist. For example, hippocampal gamma power increases may interact with alpha/beta power decreases in the visual cortex to facilitate the encoding and retrieval of visual memories (20). Speculating further, hippocampal gamma power increases may be the metaphorical spark that lights the fuse of memory replay, coded in desynchronized neocortical alpha-phase patterns (19).

Second, does the observed fast/slow gamma distinction reflect 2 true narrowband oscillations? While we have uncovered a distinction between fast and slow gamma frequencies during encoding and retrieval, we cannot say with certainty whether these differences are driven by 2 distinct oscillators, as proposed by others (31, 36, 52). Indeed, one could argue that the observed differences are driven by fluctuations in the frequency of a single oscillator. While we are unaware of such a phenomenon in hippocampal gamma, such an effect has been reported in neocortical alpha (53). Notably, however, the reported alpha-band fluctuations were very subtle (<0.5 Hz), so it would be highly questionable to interpret the much larger 25-Hz shift between fast and slow hippocampal power as originating from this alpha-band “fluctuation” mechanism. One could alternatively argue that the width of a single oscillator frequency may fluctuate as a function of memory operation, giving an apparent shift in the ratio between fast and slow gamma. However, such an effect should introduce a symmetrical change around the peak. This is not present in our data, which suggests that such an effect is ill-suited to explain the observed difference in fast and slow gamma. In short, while any electrophysiological effect can be interpreted in many ways, it seems the most parsimonious explanation here is that distinct fast and slow gamma bands differentially influence memory operations, as proposed by Colgin (7).

In summary, we demonstrate that neocortical power decreases and hippocampal power increases cooperate during the formation and retrieval of episodic memories, providing evidence that may help resolve the so-called synchronization–desynchronization conundrum (3). Furthermore, we find that distinct hippocampal gamma oscillations service human episodic memory formation and retrieval, with faster (∼60 to 80 Hz) oscillations supporting encoding and slower (∼40 to 50 Hz) oscillations supporting retrieval. In conjunction, these results further illuminate our understanding of how interactions between the neocortex and hippocampus help build and retrieve memories of our past experiences.

## Methods

### Participants.

Twelve patients (*n* = 8 from Queen Elizabeth Hospital; *n* = 4 from University Hospital Erlangen; 41.7% female; mean age, 35.5 y; range, 24 to 53 y) undergoing treatment for medication-resistant epilepsy took part in the experiment. These participants had intracranial-depth electrodes implanted for diagnostic purposes. Ethical approval was granted by the National Health Service Health Research Authority (15/WM/0219) and the Ethik-Kommission der Friedrich-Alexander Universität Erlangen-Nürnberg (142_12 B). Informed consent was obtained in accordance with the Declaration of Helsinki.

### Behavioral Paradigm: Word–Dynamic Associative Task.

Seven of the 12 participants completed this paired-associates task (Fig. 1*C*). During encoding, participants were presented with a 3-s video or sound, followed by a word in the participant’s native language (English, *n* = 7; German; *n* = 1; presented for 3 s). There was a total of 4 videos and 4 sounds, repeated throughout each block. All 4 videos had a focus on scenery that had a temporal dynamic, while the 4 sounds were melodies performed on 4 distinct musical instruments. Due to time restraints, some participants only completed the experiment using 1 modality of dynamic stimulus (sound, *n* = 1; video, *n* = 5; both, *n* = 2). Participants were asked to “vividly associate” these 2 stimuli. For each pairing, participants were asked to rate how plausible (1 for very implausible and 4 for very plausible) the association they created was between the 2 stimuli (the plausibility judgment was used to keep participants on task rather than to yield a meaningful metric). The following trial began immediately after participants provided a judgment. If a judgment was not recorded within 4 s, the next trial began. This stopped participants from elaborating further on an imagined association they had just created. After encoding, participants completed a 2-min distractor task which involved making odd/even judgments for random integers ranging from 1 to 99. Feedback was given after every trial. During retrieval, participants were presented with every word that was presented in the earlier encoding stage and, 3 s later, asked to identify the associated video/sound from a list of all 4 videos/sounds shown during the previous encoding block. The order in which the 4 videos/sounds were presented was randomized across trials to avoid any stimulus-specific preparatory motor signals contaminating the epoch. Following selection, participants were asked to rate how confident they felt about their choice (1 for guess and 4 for certain). Each block consisted solely of video–word pairs or solely of sound–word pairs; there were no multimodal blocks. Each block initially consisted of 8 pairs, with each dynamic stimulus being present in 2 trials. However, the number of pairs increased by steps of 8 if the number of correctly recalled pairs was greater than 60%—this ensured a relatively even number of hits and misses for later analysis. Participants completed as many blocks/trials as they wished. Any participant who had fewer than 10 “remembered” or 10 “forgotten” trials after intracranial (i)EEG preprocessing was excluded from further analysis.

All participants completed the task on a laptop brought to their bedside. Responses were logged using the “f,” “g,” “h,” and “j” keys, which corresponded to the values “1,” “2,” “3,” and “4.” To aid comprehension, snippets of paper were placed on top of each relevant keyboard key with the associated numerical value written upon them. The auditory stimuli were presented via the laptop’s speakers due to concerns that earphones could prove painful to the participants following electrode implantation just above the ear.

### Behavioral Paradigm: Animal–Face–Place Associative Task.

Five of the 12 participants completed this paired-associates task (Fig. 1*D*). During encoding, participants were first presented with an image cue of an animal for 2 s, followed by a pair of 2 images made up of any combination of a famous face or a famous place (i.e., face–place, face–face, or place–place pairs; presented for 2 s). There were initially a total of 20 image pairs, repeated throughout each block. This number was reduced if the hit rate fell below 66.25%, or increased if the hit rate surpassed 73.75%. Participants were asked to vividly associate these 2 stimuli. For each pairing, participants were asked whether the association was plausible or implausible (the plausibility judgment was used to keep participants on task rather than to yield a meaningful metric). Participants were self-paced in providing a judgment, and the following trial began immediately afterward. After encoding, participants completed a distractor task which involved making odd/even judgments for 15 sequentially presented random integers, ranging from 1 to 99. Feedback was given after every trial. During retrieval, participants were presented with every animal image cue that was presented in the earlier encoding stage and, 2 s later, asked how many of the associated face or place pairs they remembered (participants had the option of responding with 0, 1, or 2). If the participant remembered at least 1 image, they were then asked to select the pair of images from a panel of 4 images shown during the previous encoding block (2 targets and 2 distractors). Participants were self-paced during the retrieval stage, though the experiment ended after a runtime of 40 min in total. All participants completed the task on a laptop brought to their bedside. Any participant who had fewer than 10 remembered or 10 forgotten trials after iEEG preprocessing was excluded from further analysis.

### Behavioral Coding.

For the first associative task, trials were classified as remembered if the participant selected the correct dynamic stimulus and stated that they were highly confident about their choice (i.e., scored 4 on the 4-point confidence scale). Trials were classified as forgotten if the participant selected the incorrect dynamic stimulus, did not respond, or stated that they guessed their choice (i.e., scored 1 on the 4-point confidence scale). For the second associative task, trials were classified as remembered only if the participant indicated that they remembered both images and subsequently selected both correctly from the panel. Trials were classified as forgotten in all other cases, where the participant indicated that they did not remember at least one image and/or subsequently selected one of the images incorrectly from the panel.

### Statistical Analysis.

While the 2 tasks differed in external stimulation, the underlying cognitive and neural phenomena relating to hypotheses were expected to be consistent across tasks. Therefore, the data for the 2 tasks were pooled. Unless explicitly stated otherwise in *Results*, all statistics were conducted on the group level (i.e., random effects) using nonparametric, permutation-based statistical tests. In analyses where multiple comparisons were made (e.g., time-series differences), the false-discovery rate (FDR) correction (54) was applied (denoted *P*_fdr_). Effect sizes accompany each reported *P* value; Cohen’s *d* was used for all *t* tests (denoted *d*). For reference, Cohen (55) suggested that *d* = 0.8 indicates a large effect, *d* = 0.5 indicates a medium effect, and *d* = 0.2 indicates a small effect. Partial eta squared was used as a measure of effect size for all ANOVAs (denoted partial η^2^). For reference, partial η^2^ = 0.25 indicates a large effect, partial η^2^ = 0.09 indicates a medium effect, and partial η^2^ = 0.01 indicates a small effect.

### iEEG Acquisition and Preprocessing.

First, the raw data were epoched; for encoding trials, epochs began 2 s before the onset of the visual/auditory stimulus and ended 4 s after verbal stimulus onset (9 s in total); for retrieval trials, epochs began 2 s before, and ended 4 s after, the onset of the verbal cue (6 s in total). Second, the data were filtered using a 0.2-Hz finite-impulse response high-pass filter and 3 finite-impulse response band-pass filters at 50 ± 1 Hz, 100 ± 1 Hz, and 150 ± 1 Hz, attenuating slow drifts and line noise, respectively. Third, as the iEEG data were sampled at the physician’s discretion (512 Hz, *n* = 1; 1,024 Hz, *n* = 8), all data were downsampled to 500 Hz. Fourth, the data from each electrode were rereferenced to an electrode on the same shaft that was positioned in white matter (determined by visual inspection of participant anatomy; see below). The use of a common reference electrode for both the hippocampus and neocortex ensured that any difference in electrophysiological signal from the 2 regions could not be explained by a difference in reference. Finally, the data were visually inspected and any trials exhibiting artifactual activity were excluded from further analysis. Any electrodes exhibiting persistent ictal and interictal activity (as identified through visual inspection) were discarded from analysis.

### Electrode Localization.

First, hippocampal and white matter contacts were defined based on anatomical location through visual inspection of the T1-weighted anatomical scan (nb., one participant had no hippocampal contacts, and therefore was excluded from all hippocampal-based analyses). Then, the native space coordinates of all remaining contacts were determined by visual inspection of each participant’s postimplantation T1 scan. These contact coordinates were then transformed from native space to Montreal Neurological Institute (MNI) space using a transform matrix obtained by normalizing participant T1 scans in SPM 12. These contacts were then marked as within the anterior temporal lobe or elsewhere (this latter group was excluded from further analysis). The ATL was defined as all parts of the temporal lobe [as defined by the *wfupickatlas* plugin (56) for SPM 12] anterior to a plane perpendicular to the long axis of the temporal lobe (57). The plane was slightly shifted from that described in ref. 57 to [*y* = −5, *z* = −30; *y* = 15, *z* = −5] for the pragmatic reason of ensuring that all participants had electrode contacts in the ATL ROI. For visualization in Fig. 1*D*, every electrode from every participant was given a diameter of 1 cm and then placed in a template brain registered in MNI space. The number of electrodes in each voxel was then summed to provide a measure of summed density.

### 1/*f* Correction.

Spectral power was computed using 199 linearly spaced 5-cycle wavelets ranging from 1 to 100 Hz. The time–frequency decomposition method was kept consistent across all frequency bands to ensure that only a single slope (characterizing the full extent of the 1/*f* dynamic) needed to be calculated and subsequently subtracted from the signal (in line with previous experiments that have extracted the 1/*f* characteristic from the signal [e.g., refs. 39 and 40]). A vector containing values of each wavelet frequency (*A*) and another vector containing the power spectrum for each electrode–sample pair (*B*) were then log-transformed. The linear equation *Ax = B* was solved using least-squares regression, where *x* is an unknown constant describing the curvature of the 1/*f* characteristic. The 1/*f* fit (*Ax*) was then subtracted from the log-transformed power spectrum (*B*).

### Peak Frequency Analysis.

Raw signal recorded at every contact for each epoch was convolved with a 5-cycle wavelet (0 to 1,500 ms poststimulus [padded with real data for lower frequencies], in steps of 25 ms; 1 to 100 Hz, in steps of 0.5 Hz). The 1/*f* noise was subtracted using the method described above to help pronounce the peaks in the power spectrum. The data were then smoothed using a Gaussian kernel (full-width half-maximum, 200 ms; 1 Hz) to attenuate inter- and intraindividual differences in spectral responses (53) and to help approximate normally distributed data (an assumption frequently violated in small samples). The data were averaged across all time points, trials, and contacts (separately for the hippocampus and ATL). Peaks of 1/*f*–corrected absolute power were then identified using the *findpeaks()* peak-detection algorithm implemented in MATLAB. To identify the memory-related difference in the dominant gamma bands, the power spectra for remembered trials were calculated in an identical manner, except that the Gaussian kernel was expanded to account for the greater variability of high-frequency oscillatory responses (200 ms, 5 Hz). The power spectra for encoding and retrieval were then collapsed into seven 10-Hz bins ranging from 30 to 100 Hz and contrasted in a group-level (i.e., random effects), nonparametric permutation test (58) with 5,000 randomizations. The multiple-comparisons issue was solved using the false-discovery rate correction (54). This analysis was repeated for the forgotten trials.

#### Selection of peak frequencies.

The peak frequencies of each patient were determined using the MATLAB function *findpeaks()* on the averaged power spectrum around the approximate frequency bands (theta, 1 to 7 Hz; alpha/beta, 8 to 20 Hz; slow gamma, 30 to 60 Hz; fast gamma, 50 to 100 Hz). The bandwidths of these peaks were kept consistent across participants, and were determined through inspection of the group-averaged bandwidth of the peaks (theta, ±0.5 Hz; alpha/beta, −1 Hz/+5 Hz [capturing the observed asymmetry in the peak]; slow/fast gamma, ±10 Hz). Individual peak frequencies are reported in *SI Appendix*, Table S1.

### Spectral Power Analysis.

For all spectral power analyses (i.e., encoding and retrieval epochs), the data underwent the same wavelet convolution, 1/*f* correction, and smoothing approaches described in *Peak Frequency Analysis*. The data were then *z*-transformed using the means and SDs of each electrode–frequency pair (14). The time–frequency–resolved data were then averaged over electrodes of each ROI. For time-series statistical analysis, trials were split into 2 groups based on whether the stimuli were remembered or forgotten. Then, the time series were collapsed into 7 time bins of 200 ms and the 2 conditions were contrasted using the same nonparametric statistical procedure described in *Peak Frequency Analysis*. For statistical analyses of the interaction between memory task (encoding vs. retrieval) and gamma frequency (fast vs. slow), this memory-related difference in power (i.e., SME and RSE) was averaged over time and contrasted in a nonparametric, permutation-based, 2 × 2 repeated-measures ANOVA.

### Cross-Correlation Analysis.

For all cross-correlation analyses (i.e., encoding and retrieval epochs), the data underwent the same wavelet convolution, 1/*f* correction, and smoothing approaches described in *Spectral Power Analysis*, with 2 exceptions: 1) wavelet convolution occurred in steps of 10 ms rather than 50 ms (enhancing temporal resolution), and 2) the temporal aspect of the smoothing kernel was reduced to 50 ms to avoid excessive smoothing obscuring the temporal dynamics of the neocortical–hippocampal cross-correlation. For each “trial × electrode combination” pair, the cross-correlation between the hippocampus and ATL was computed using the MATLAB function *crosscorr()* with a lag of 300 ms (meaning the correlation between the hippocampus and neocortex was considered for every offset from where the neocortex preceded the hippocampus by 300 ms to where the neocortex lagged behind the hippocampus by 300 ms). This returned a time series of Pearson correlation values describing the relationship between the hippocampus and neocortex at all considered lags. These correlation values were then averaged over electrodes and split into 2 groups: remembered and forgotten. These 2 groups were individually averaged over trials for each participant, collapsed into bins of 100 ms, and then contrasted using the same nonparametric statistical procedure described in *Peak Frequency Analysis*. We term the “remembered > forgotten” difference in cross-correlation for encoding data the “subsequent memory cross-correlation” and the difference for retrieval data the “retrieval success cross-correlation.”

To test the “encoding–retrieval” × “lag–lead” difference, we contrasted the subsequent memory cross-correlation with the retrieval success using the same nonparametric statistical procedure described in *Peak Frequency Analysis*.

Lastly, to test the influence of the “memory task” × “gamma frequency” interaction on the memory-related cross-correlation differences, we conducted a nonparametric, permutation-based, 2 × 2 repeated-measures ANOVA in the same manner as described in *Spectral Power Analysis*.

## Supplementary Material

Supplementary File
