# Supplementary material for: Directional coupling of slow and fast hippocampal gamma with neocortical alpha/beta oscillations in human episodic memory
Source: Proc Natl Acad Sci U S A. 2019 Oct 9;116(43):21834–42. doi: 10.1073/pnas.1914180116 (PMC6815125; doi:10.1073/pnas.1914180116)
Supplement: Supplementary File [file pnas.1914180116.sapp.pdf]

SUPPLEMENTARY INFORMATION

Supplementary Results

Event-related potentials

Measures of low-frequency oscillatory power (<10Hz) can be distorted by event-related potentials (ERPs). To rule out the concern that the observed memory-related power effects are driven by ERPs, the ERPs themselves were examined. The data was epoched from 500ms pre-stimulus to 1000ms post-stimulus and high frequency activity was filtered out using a Butterworth low-pass filter of 20Hz. Baseline correction was computed as the difference in amplitude from a pre-stimulus window (-250 to 0ms). Trials were split based on whether the stimulus pairs were remembered or forgotten, and the data was then averaged across contacts of the same region of interest. Remembered trials were contrasted with forgotten items in a non-parametric, group-level t-test. At encoding, no memory-related difference in ERPs was observed in the ATL (p_{fdr} = 0.178) or hippocampus (p_{fdr} = 0.368). Similarly during retrieval, no memory-related difference in ERPs was observed in the ATL (p_{fdr} = 0.291) or hippocampus (p_{fdr} = 0.279).

The influence of reference on cross-correlation results

The choice of reference can have a drastic impact on cross-regional analysis such as the cross-correlation result reported in the main text. Indeed, it is possible that as neocortical and hippocampal electrodes on the same shaft shared a common (white matter) reference, an artificial correlation between two regions may arise as a result of shared reference activity. However, such a correlation for power can only be positive and zero centred which is in stark contrast to the negative non-zero centred correlation reported in the main text. In the main text, this potential confound was tackled by contrasting hits with misses to subtract out any reference-related confound (as both conditions should fall foul to the issue) while preserving statistical power. Here, we complimented the subtraction approach by reproducing the cross-correlation analysis using different references for hippocampal and neocortical electrodes. The analytical approach is identical to that reported in the main text with one key exception: when creating hippocampal-neocortical electrode pairs, any pair from the same shaft (i.e. shared the same white matter reference) was excluded from analysis (leaving only electrode pairs with different white matter reference). Notably, one participant had only a single shaft where the hippocampus and ATL was recorded from, meaning that all hippocampal and ATL recordings in this participant shared a white matter reference. This participant was therefore excluded from this analysis. This approach yielded near identical results to those reported in the main text. ATL alpha/beta power decreases preceded hippocampal fast gamma power increases during successful memory formation (p_{fdr} = 0.026, d = 0.954), while hippocampal slow gamma power increases preceded ATL alpha/beta power decreases during successful memory retrieval (p_{fdr} = 0.035, d = 0.741). Similarly, the 2 x 2 (gamma frequency x encoding/retrieval) was replicated (p = 0.013, partial eta squared = 0.136). These findings suggest that the cross-correlation results are not due to spurious correlations owing to the choice of referencing.

The influence of interictal discharges on cross-correlation results

Notably, epileptic activity such as IEDs (Inter-Epileptical Discharges) can travel between the neocortex and the hippocampus, and these discharges can influence whether a memory is encoded/retrieved or not. While the data was cleaned of these discharges, a control analysis was run where any electrodes near the seizure foci (as described in supplementary table 4) were excluded. It is important to note

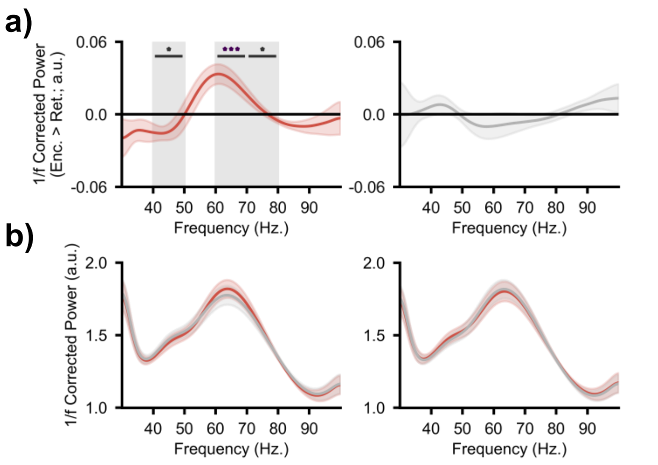

Supplementary Figure 1. Encoding-retrieval differences in hippocampal gamma. **(a)** the difference in encoding hippocampal gamma power and retrieval hippocampal gamma power for remembered items (left) and forgotten items (right). **(b)** the power spectra for encoding and retrieval hippocampal gamma power for remembered items (left) and forgotten items (right). A clear "fast" gamma peak can be seen between 60 and 70Hz. A second notch can be seen between 40 and 50Hz, reflecting "slow" gamma activity.

that only seven subjects maintained electrodes in both the ATL and the hippocampus, and therefore were included in this control analysis.

For this approach, the memory-related alpha-gamma contrast in the ATL at encoding and at retrieval continued to demonstrate a statistically significant effect (p_{fdr} = 0.041, Cohen's d = 0.485, and p_{fdr} = 0.036, Cohen's d = 1.302). Similarly, the encoding-retrieval contrast within the ATL matched those reported in the main text (-100 to -200ms, $p_{fdr} < 0.001$, Cohen's d = 1.104; 200 to 300ms, $p_{fdr} < 0.001$, Cohen's d = 1.647). Lastly, the 2 x 2 (gamma frequency x encoding/retrieval) continued to reveal a significant interaction (p = 0.036, partial eta squared = 0.234). Together, these results analytically demonstrate that interictal discharges cannot explain the observed results.

Supplementary Discussion

On the "fast"/"slow" gamma distinction: Notably, the raw power spectra (see supplementary figure 1b) do not show two clear gamma peaks, but rather a singular peak around the "fast" gamma frequency, with a smaller "slow" gamma notch on the rising slope of the gamma peak. While this may speak against the idea that two distinct gamma band oscillators differentially influence encoding and retrieval, such an effect has been reported in rodents before. Bragin and colleagues[1] demonstrated that "fast" gamma oscillations can effectively mask "slow" gamma oscillations, and the "fast" oscillator must be lesioned to observe the "slow" gamma oscillations. While lesioning would be highly unethical in humans, directly contrasting the power spectra at encoding and retrieval produces a synonymous result because it subtracts the amplitude imbalance in "fast" and "slow" gamma power common across all trials and returns the residual difference (see figure 2b). In other words, the gamma-band raw power spectra can be misleading due to masking of "slow" gamma by high-amplitude "fast" gamma, but 'encoding versus retrieval' contrasts address these issues.

References

1. Bragin, A. *et al.* Gamma (40-100 Hz) oscillation in the hippocampus of the behaving rat. *J. Neurosci.* **15**, 47–60 (1995).

2. Hanslmayr, S., Staresina, B. P. & Bowman, H. Oscillations and episodic memory – Addressing the synchronization/desynchronization conundrum. *Trends Neurosci.* **39**, 16–25 (2016).

3. Hanslmayr, S., Staudigl, T. & Fellner, M.-C. Oscillatory power decreases and long-term memory: the information via desynchronization hypothesis. *Front. Hum. Neurosci.* **6**, 1–12 (2012).

4. Colgin, L. L. & Moser, E. I. Gamma oscillations in the hippocampus. *Physiology* **25**, 319–329 (2010).

5. Staresina, B. P. *et al.* Hippocampal pattern completion is linked to gamma power increases and alpha power decreases during recollection. *Elife* **5**, 1–18 (2016).

Supplementary Table 1. Resonating frequencies of each patient (1-7 took part in task 1; 8-12 took part in task 2).

	Neocort. Alpha/Beta	Hipp. "Slow" Gamma	Hipp. "Fast" Gamma
Patient 1	12.5	39.5	64.5
Patient 2	10.0	38.0	56.0
Patient 3	11.5	49.0	63.0
Patient 4	12.0	44.5	66.0
Patient 5	14.5	44.5	67.0
Patient 6	16.0	46.5	68.0
Patient 7	10.0	45.5	70.0
Patient 8	10.5	47.5	71.0
Patient 9	10.0	40.0	56.0
Patient 10	10.5	44.5	63.5
Patient 11	10.5	47.0	60.5
Patient 12	10.0	38.5	64.5
**Mean**	**11.5**	**43.8**	**64.2**

Supplementary Table 2. Number of electrodes in each region of interest per participant (1-7 took part in task 1; 8-12 took part in task 2).

	Hippocampus	Anterior Temporal Lobe	Total
Patient 1	3	3	6
Patient 2	3	3	6
Patient 3	1	4	5
Patient 4	4	3	7
Patient 5	1	3	4
Patient 6	2	3	5
Patient 7	2	3	5
Patient 8	3	2	5
Patient 9	2	2	4
Patient 10	2	3	5
Patient 11	2	7	9
Patient 12	2	3	5
**Total**	**27**	**39**	**66**
**Median**	**2**	**3**	**5**

Supplementary Table 3. Number of trials per condition, per participant following artefact rejection (1-7 took part in task 1; 8-12 took part in task 2).

	Encoding			Retrieval		
	Remembered	Forgotten	Total	Remembered	Forgotten	Total
Patient 1	14	48	62	15	62	77
Patient 2	19	19	38	22	26	48
Patient 3	30	18	48	35	21	56
Patient 4	31	26	57	37	24	61
Patient 5	31	29	60	36	31	67
Patient 6	90	52	142	91	51	142
Patient 7	21	12	33	21	16	37
**Mean**	**33.7**	**29.1**	**62.9**	**36.7**	**33.0**	**69.7**
Patient 8	63	94	157	69	93	162
Patient 9	28	22	50	45	28	73
Patient 10	113	32	165	144	40	184
Patient 11	173	48	221	186	18	204
Patient 12	147	70	217	145	69	214
**Mean**	**104.8**	**53.2**	**162.0**	**117.8**	**49.6**	**167.4**

Supplementary Table 4. Additional patient demographics (1-7 took part in task 1; 8-12 took part in task 2).

Patient No.	Years of Seizures	Dominant Hand	Epileptic Foci
Patient 1	17	Right	Left hippocampus
Patient 2	3-5	Right	Bilateral medial temporal lobe
Patient 3	34	Unknown	Left frontal lobe
Patient 4	9-12	Right	Bilateral medial temporal lobe
Patient 5	8	Right	Right neocortical temporal lobe
Patient 6	10-12	Left	Right medial parietotemporal lobe
Patient 7	26	Right	Right medial temporal lobe
Patient 8	Unknown	Right	Unknown
Patient 9	26	Right	Right medial temporal lobe
Patient 10	Unknown	Right	Unknown
Patient 11	Unknown	Left	Left frontal lobe
Patient 12	22	Right	Left medial temporal lobe
